# Effect of a very low negative dietary cation-anion difference (DCAD) diet on plasma and urine metabolomics of prepartum Holstein cows

**DOI:** 10.3168/jdsc.2021-0154

**Published:** 2021-10-22

**Authors:** P. Melendez, P.K. Chelikani, D. Patel, E. Garbarino

**Affiliations:** 1 School of Veterinary Medicine, Texas Tech University, Amarillo 79106; 2 Health Science Center, Texas Tech University, Amarillo 79106; 3 Progressive Dairy Health Service, Clovis, NM 88101

## Abstract

The objectives of this cross-sectional, nonintervention, observational study were to compare urine and blood parameters between cows consuming a positive dietary cation-anion difference (DCAD) diet [early dry cows, DCAD + 250 mEq/kg of dry matter (DM), n = 15] with the same cows consuming a negative DCAD diet (−220 mEq/kg of DM) 10 d after moving them from the early dry to the prepartum group. The most remarkable finding was that cows consuming the anionic diet had very low urine pH and very low base excess in blood, suggestive of uncompensated metabolic acidosis. Importantly, the metabolomics data revealed that only urine concentrations of essential and aromatic amino acids were decreased, and that concentrations of total nonessential amino acids and glucogenic amino acids were increased in plasma and reciprocally decreased in urine, suggesting that the cows fed anionic salts were attempting to meet a high glucose demand by mobilizing gluconeogenic amino acid reserves. Notably, the dietary anionic salts exerted marked effects on glycerophospholipids, with a reduction in most phosphatidylcholine containing diacyl (PC aa) and acyl-alkyl (PC ae) moieties in plasma and urine. Further characterization of these metabolomic profiles may lead to the development of novel biomarkers to identify cows susceptible to metabolic acidosis and other metabolic diseases.

## Highlights

• Cows consuming a very low negative DCAD diet (-220 mEq/kg) had very low urine pH (range: 5.0–5.8).
• Blood parameters of cows consuming a very low negative DCAD diet showed high levels of lactate, low levels of bicarbonate, and very low base excess.
• Metabolomics of negative DCAD diets showed high levels of total NEAA and glucogenic AA in plasma and decreased levels in urine, and reduced PC aa and PC ae moieties in plasma and urine.

## Graphical Abstract

**Figure:**
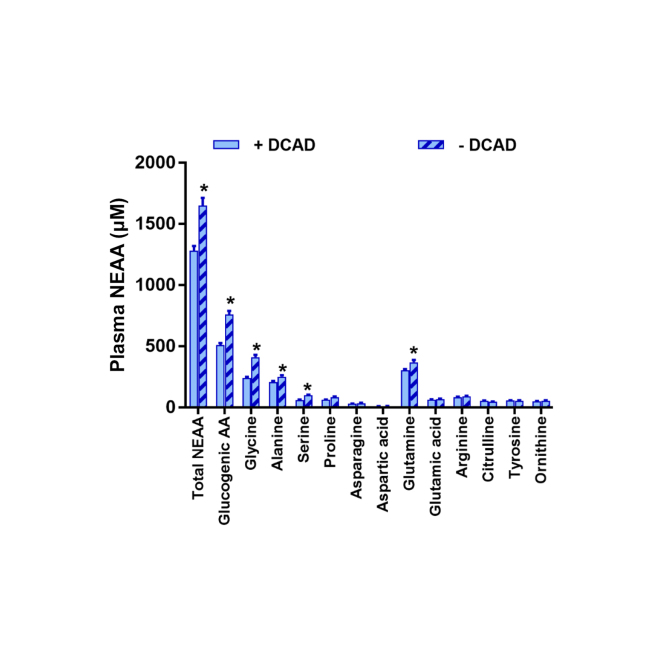
**Summary:** Cows consuming a very low negative DCAD diet had urine pH <6.0 and blood base excess less than -6, suggestive of uncompensated metabolic acidosis. Metabolomics data revealed that only urine concentrations of essential and aromatic amino acids were decreased, and that concentrations of total nonessential amino acids and glucogenic amino acids were increased in plasma and reciprocally decreased in urine. The anionic diet markedly reduced phosphatidylcholine containing diacyl (PC aa) and acyl-alkyl (PC ae) moieties in plasma and urine.

Hypocalcemia is a common metabolic disorder affecting dairy cattle. Subclinical hypocalcemia affects nearly half of postpartum dairy cows and increases their susceptibility to other metabolic and infectious diseases (Goff, 2014; Melendez and Risco, 2016). Among the various prevention strategies for hypocalcemia, the most popular includes the use of anionic compounds in diets to alter the differences in milliequivalents between potassium and sodium (cations) and chloride and sulfates (anions) (DCAD). Cows are typically fed diets with high DCAD due to high K content, which places the cow in a state of compensated metabolic alkalosis. In this state, tissue responsiveness to parathyroid hormone (PTH) may be reduced, which leads to impaired Ca homeostasis (Goff, 2014). Anions added to the diet can restore parathyroid hormone sensitivity by reducing the degree of alkalosis of the cow (Goff, 2014, 2018; Vieira-Neto et al., 2021a); consequently, enhanced vitamin D activity, and better Ca resorption from the bone occurs (Goff, 2018; Lean et al., 2019). However, as more anions are fed, H^+^ in the blood increases and pH decreases, leading to a potential uncompensated metabolic acidosis. Acidosis can be measured through urine pH, but the ideal pH level for the prevention of milk fever has been very controversial. Urinary pH values below 6.0 may indicate a risk of overacidification and development of a potential uncompensated metabolic acidosis (Charbonneau et al., 2006; Goff, 2018). Urinary pH below 7.0 has been consistently related to a lower incidence of milk fever (Charbonneau et al., 2006). Consequently, it has been recommended that the optimal urinary pH for prepartum dairy cows should be between 6.0 and 7.0 for Holstein cows (Charbonneau et al., 2006; Melendez and Poock, 2017; Melendez et al., 2021). Several nutritional approaches in the US dairy industry and other countries are focused on reducing the urinary pH to as low as 5.5. By reducing the pH from normal values of 8.5 to values close to 5.5 (3 pH units), an excessive load is imposed on the kidneys, as they must excrete 1,000 times the additional H^+^ produced by the body (Goff, 2018). The impact of anionic diets on the metabolic status of both the cows and their calves is not well understood, and such levels of overacidification may have detrimental effects on both the dam and her offspring.

We hypothesized that high levels of anionic salts would increase urine acidity, promote metabolic acidosis, and alter metabolomic profiles in the plasma and urine of prepartum Holstein cows; consequently, this cross-sectional study aimed to evaluate the association of urine pH (<6.0) and blood acid-base parameters and metabolomics of plasma and urine in prepartum Holstein cows fed an anionic diet with very low DCAD.

The study was conducted at a commercial Holstein dairy farm located in the southwest Texas panhandle (Amherst, TX). The dairy milked approximately 2,600 Holstein cows 3 times a day, with a rolling herd average of 13,505 kg/cow. The study was approved by the Institutional Animal Care and Use Committee of the Texas Tech University (IACUC protocol ID: 21045–05).

This research was a nonintervention observational study where prepartum cows were fed an anionic diet with very low DCAD (−220 mEq/kg of DM). There was no prepartum control group with a positive DCAD diet. Therefore, the same cows served as their own control, which allowed for comparison of their metabolic and acid-base status before (positive DCAD) and after (negative DCAD) entering the prepartum group. For this purpose, a first visit to the farm was conducted on May 7, 2021, to select cows with urine pH <6.0 and >6.0. After sampling the first 10 cows (visit 1), the urine pH results unexpectedly showed that all were <6 (data not shown). Hence, a further sampling (visit 2) was done on June 9, 2021, to sample a different group of cows (range of days to parturition: 21 to 30). Dry cows consuming a regular diet (without anionic products; Table 1) before starting their prepartum period comprised the target population. To find a difference in urine pH of 1.5 units (SD = 0.8) between dry cows before entering the prepartum group and the same cows 10 d after entering the prepartum group with 99% confidence and 90% power, a sample size of 9 cows was calculated. To ensure that at least 9 cows would be collected for urine on a further visit 10 d after starting to consume the anionic diet, a group of 16 dry cows fed a diet with a positive DCAD (+250 mEq/kg) immediately before entering the prepartum lot (21 to 30 d before expected parturition) were randomly selected at once (on the same day) and sampled for urine pH and blood. Ten days after moving the cows to the prepartum lot (receiving a diet with a DCAD −220 mEq/kg), they were sampled for urine and blood for a second time (visit 3) to compare metabolic variables between the feeding periods (positive vs. negative DCAD). During the second visit, an early dry cow and a prepartum diet sample were obtained from the feed bunk immediately after feeding a newly prepared mix to the group. Samples were submitted to a commercial laboratory (Dairyland Laboratories Inc., Arcadia, WI) for near infrared spectroscopy (NIRS) analysis. Unfortunately, it was not possible to obtain a proper urine sample from 1 cow out of 16; therefore, the urine and blood samples of only 15 cows were analyzed and compared between the 2 periods. At assignment, days to expected parturition and parity were recorded. Body condition score was assessed using a scale from 1 to 5 in quarter-unit increments (Ferguson et al., 1994).

**Table 1:** Ingredients and nutrient composition of early dry and prepartum cow diets during visit 2 (June 9, 2021)^1^

Item	Early dry	Prepartum
Ingredient (kg of DM)		
Wheat hay	2.72	2.50
Wheat straw	2.27	—
Wheat silage	3.86	—
Sorghum silage	4.77	0.90
Corn silage	—	4.86
Corn gluten feed	0.68	—
Soybean meal	0.59	0.81
Corn grain flaked	—	3.40
Mineral-vitamin premix^2^	0.11	0.66
Total weight (kg)	15.00	13.13
Nutrient composition (% of DM)		
DM	42.55	50.90
CP	10.59	14.21
aNDFom^3^	59.91	34.33
Starch	7.86	16.55
Sugars	4.04	4.58
Fat (ether extract)	2.80	3.05
Ash	7.59	9.14
Ca	0.65	1.02
P	0.38	0.41
Mg	0.25	0.41
K	1.82	1.15
S	0.18	0.24
Na	0.08	0.026
Cl	0.49	1.34
DCAD^4^	+250	−220

1 Diet samples were submitted to Dairyland Laboratories Inc. (Arcadia, WI) for near infrared spectroscopy analysis.

2 Anionic premix for prepartum cows.

3 aNDFom = amylase neutral detergent fiber organic matter.

4 (Na + K) − (Cl + S); mEq/kg of DM.

A clean-caught, uncontaminated, mid-stream urine sample was obtained by gentle massage of the escutcheon area and placed in plastic containers. Urine pH was determined immediately by a portable meter (Hanna Instruments), and retested at the Texas Tech University laboratory (Amarillo). Blood samples were obtained from the tail vein plexus using a Vacutainer system (Becton, Dickinson and Co.) with heparin and tested within ~1 h of collection from all cows for pH, partial pressure of CO_2_ and O_2_ (mm Hg), total CO_2_ (mmol/L), saturated O_2_ (%), bicarbonate (HCO_3_^−^; mmol/L), lactate (mmol/L), and base excess (mmol/L) using a portable meter (i-Stat, Abbott). The rest of the blood was collected and placed in an ice chest and centrifuged within 2 h at 1,500 × *g* for 15 min, and plasma was separated in centrifuge tubes. Plasma and aliquots of urine were frozen on dry ice, transported to the laboratory, and stored at −80°C. Immediately after arrival at the laboratory, urine samples were retested for pH assessment to corroborate the on-farm evaluation, using an electronic pH meter (model HI5521, Hanna Instruments). However, a limitation was that urine specific gravity and total urine output were not measured.

Plasma and urine samples from all visits were analyzed using a commercially available Targeted Metabolomics kit (Absolute IDQ p180, Biocrates Life Sciences AG). The kit was developed and validated to measure up to 188 endogenous, nutrient-derived, and microbiome-derived metabolites across 6 analyte classes. These analyte classes include 21 AA, 21 biogenic amines, 1 monosaccharide, 40 acylcarnitine, 90 glycerophospholipids, and 15 sphingomyelins. Sample preparation was performed as per the kit's user manual. Briefly, the samples (10 μL) were first loaded on the 96-well filter plate and dried under nitrogen using a nitrogen evaporator. The dried samples were derivatized using phenylisothiocyanate (PITC) reagent in the presence of ethanol, water, and pyridine at room temperature for 30 min to derivatize AA and biogenic amine metabolites. Next, the samples were extracted with 300 µL of 5 m*M* ammonium acetate in methanol using a positive pressure manifold and collected into a lower 96-well collection plate. The extracted metabolites were analyzed using 2 techniques. First, AA and biogenic amines were separated by reverse-phase chromatography using ultra-high-performance liquid chromatography (UHPLC, Nexera-LC-30 series, Shimadzu) and detected in positive mode by a tandem mass spectrometer (5500 QTRAP, Sciex). A 7-point calibration curve for each analyte was used to quantify AA and biogenic metabolites. Second, acylcarnitines, glycerophospholipids, sphingolipids, and hexose sugars were analyzed in positive ionization by flow injection analysis using liquid chromatography-tandem MS (LC-MS/MS; 5500 QTRAP, Sciex). Each of the targeted metabolites was identified and quantified using their mass-to-charge ratio (*m*/*z*) or multiple reaction monitoring transition and retention time. Peak identification and confirmation were performed using Analyst software (Sciex) and then transferred to MetIDQ (Biocrates Life Sciences AG) software for data analysis and calculation.

Urine pH and blood metabolites were analyzed by a one-way repeated-measures ANOVA for paired samples using PROC GLM of SAS Institute (2017). The model included period of feeding (early dry vs. prepartum), BCS (range: 2.75 to 3.75), parity (8 cows going to a second lactation, 6 cows to third lactation, and 1 cow to fourth lactation), and days to expected parturition (range: 21 to 30 d). For metabolite classes, the differences between groups were compared using Benjamini-Hochberg correction with a false discovery rate of 0.05 (Benjamini and Hochberg, 1995) using Graph Pad 7.0 (GraphPad Inc.). Further metabolomic analyses were conducted using Metaboanalyst 5.0 (Pang et al., 2021). Briefly, a hierarchical clustering heatmap was generated using the Ward algorithm for the top 25 metabolites ranked by *t*-tests and ANOVA. A sparse partial least squares discriminant analyses (**sPLS-DA**) was used to reduce the total variables to 2 components with 10 metabolites per component. Significance and tendency levels were declared at *P* ≤ 0.05 and *P* ≤ 0.1, respectively.

Urine pH and blood analytes for the early dry and prepartum periods (visits 2 and 3), respectively, were as follows: urine pH: 8.18 and 5.33 (SEM = 0.20; *P* ≤ 0.0001); blood pH: 7.50 and 7.36 (SEM = 0.048; *P* ≤ 0.0001); base excess: 2.46 and −7.79 mmol/L (SEM = 1.75; *P* ≤ 0.0001); lactate: 0.99 and 1.49 mmol/L (SEM = 0.55; *P* ≤ 0.05); HCO_3_^−^: 25.65 and 17.45 mmol/L (SEM = 1.51; *P* ≤ 0.0001); saturated O_2_: 68.73 and 52.0% (SEM = 16.3; *P* ≤ 0.01); total CO_2_: 26.59 and 18.39 mmol/L (SEM = 1.63; *P* ≤ 0.0001); partial pressure of CO_2_: 32.62 and 30.68 mm Hg (SEM = 4.39; *P* > 0.05); and partial pressure of O_2_: 37.51 and 29.85 mm Hg (SEM = 18.52; *P* > 0.05). Effects of parity, BCS, and days to parturition for all outcome variables were not significant (*P* > 0.05). The intra-class correlation coefficient between on-farm urine pH and laboratory urine pH was 0.99, meaning the on-farm evaluation was an accurate method by which to determine urine pH under field conditions.

One of the weaknesses of this study was the lack of a positive DCAD control group during the prepartum period to compare animals with the same length of pregnancy with and without consuming an anionic diet. We acknowledge that days of gestation at sampling is a confounder for the outcome variables of this study, such that the early dry cows had fewer days of pregnancy than the same cows sampled 10 d after the first testing. However, the alternative of having a control group without anionic salts was not feasible because the study was carried out on a commercial farm, without the possibility of intervention, because a control group with a positive DCAD could put the animals at risk of developing milk fever. Further, in the statistical model (repeated-measures one-way ANOVA for paired samples), days of gestation of the cows at the first and second samplings were included as a covariate.

Cows consuming the anionic diet had a very low urine pH, ranging from 4.96 to 5.74, and a very low base excess in blood. The metabolomics data revealed that after 1 wk of transitioning to the negative DCAD diet, EAA concentrations were unaltered in plasma but decreased in the urine, along with a reduction in urine concentrations of aromatic AA, and histidine, lysine, and threonine (Figure 1A, I). These findings suggest that the demand for EAA was likely higher on the negative DCAD diet and cows were attempting to prevent urinary losses to conserve circulating concentrations. Interestingly, concentrations of total NEAA and glucogenic AA increased in plasma (Figure 1B) and reciprocally decreased in urine (Figure 1J) following feeding of anionic salts. Whereas glycine, alanine, and glutamine were increased in plasma, alanine, glutamine, and glutamic acid were decreased in the urine of cows fed a negative DCAD diet (Figure 1B, J). Together, these findings suggest that the cows fed anionic salts were attempting to meet a high glucose demand by mobilizing gluconeogenic AA reserves. The biogenic amines were unaltered in plasma but urine concentrations of creatinine, α-aminoadipic acid, and taurine were reduced by the negative DCAD diet.

**Figure 1:**
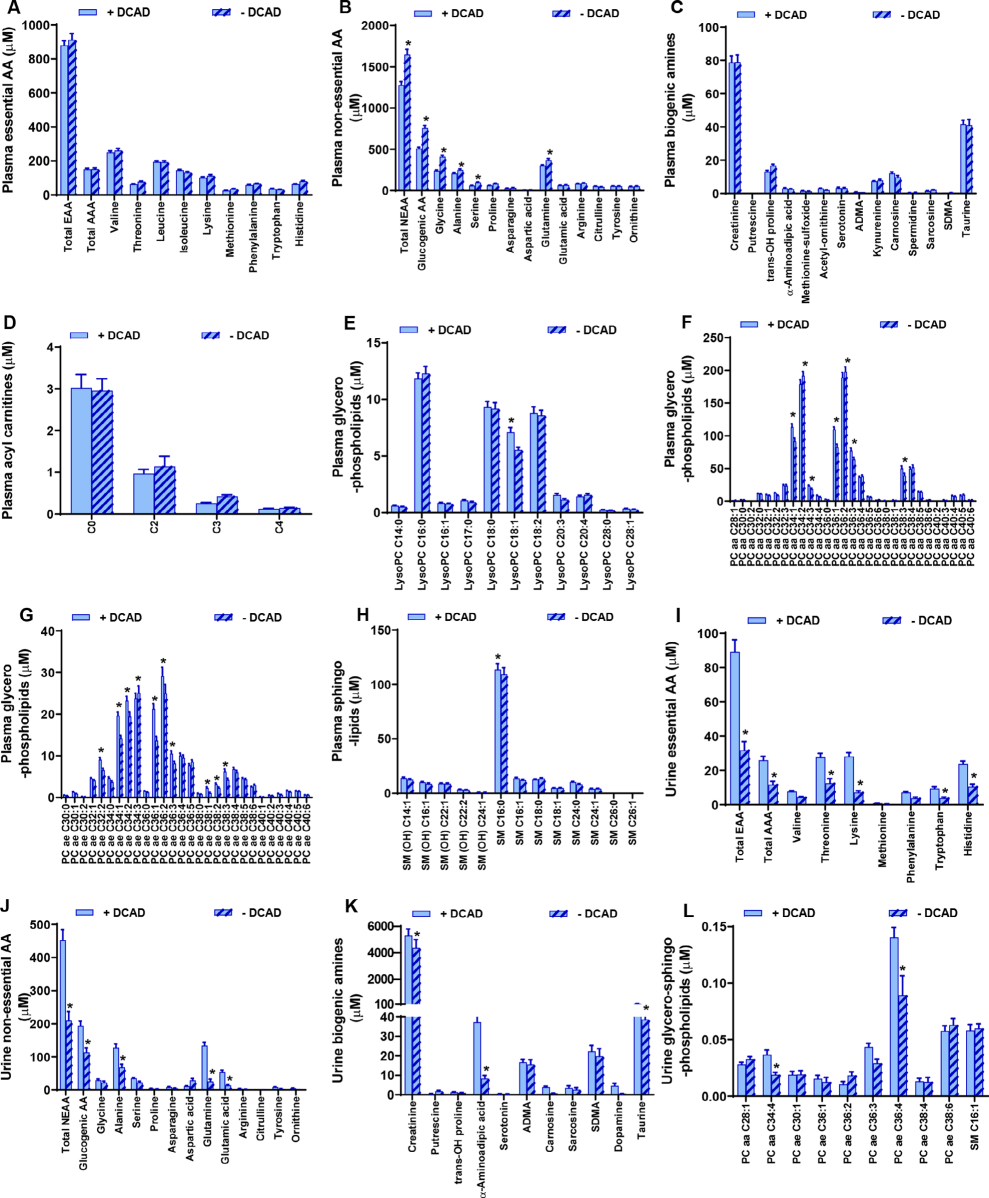
Effects of early dry DCAD (+220 mEq/kg of DM) and prepartum DCAD (−250 mEq/kg of DM) diets fed to pregnant cows (visits 2 and 3), on targeted metabolomics in plasma and urine. Panels show plasma concentrations of (A) EAA, (B) NEAA, (C) biogenic amines, (D) acyl carnitines, (E, F, G) glycerophospholipids, and (H) sphingolipids. Also shown are urine concentrations of (I) EAA, (J) NEAA, (K) biogenic amines, and (L) glycerophospholipids and sphingolipids. Each point represents mean ± SEM; *P ≤ 0.05. AAA = α-aminoadipic acid; ADMA = asymmetric dimethylarginine; SDMA = symmetric dimethylarginine; LysoPC = lysophosphatidylcholine; PC aa = phosphatidylcholine with diacyl residue sum; PC ae = phosphatidylcholine with acyl-alkyl residue sum; SM (OH) = hydroxysphingomyelin with acyl residue sum; SM = sphingomyelin with acyl residue sum.

Concentrations of acyl carnitine were unaltered in plasma (Figure 1D) and undetectable in urine; with the exception of a reduction in plasma lysophosphatidylcholine with acyl residue C18:1 in plasma by the anionic diet, the rest were unaltered (Figure 1E). The dietary anionic salts exerted marked effects on glycerophospholipids, with reductions in phosphatidylcholine with diacyl residue sum (**PC aa**) moieties: PC aa C34:1, PC aa C34:3, PC aa C36:1, PC aa C36:3, and PC aa C38:3, and increases in PC aa C34:2 and PC aa C36:2 in plasma and PC aa C28:1 in urine (Figure 1F, J). The anionic diets decreased plasma concentrations of several phosphatidylcholine with acyl-alkyl residue sum (**PC ae**) moieties: PC ae C32:2, PC ae C34:1, PC ae C34:2, PC ae C34:3, PC ae C36:1, PC ae C36:2, PC ae C36:3, PC ae C38:1, PC ae C38:2, and PC ae C38:2 (Figure 1G), and some moieties (PC aa C34:4 and PC ae C36:4) were decreased in urine (Figure 1L). Consistent with our findings of reductions in circulating long-chain phosphatidylcholine moieties, others have noted similar reductions of some phosphatidylcholines in skeletal muscle (PC ae C34:3, PC ae C36:3, PC ae C38:3) of prepartum cows with high BCS (Sadri et al., 2020), in urine (PC ae C34:1) of prelame cows (Eckel et al., 2020), and in plasma (PC aa C36:3, PC aa C38:3, PC ae C34:1, PC ae C36:2, PC ae C36:3, PC ae C38:2) of cows with hepatic lipidosis (Imhasly et al., 2014). However, unlike the effects of negative DCAD diet in our study, plasma concentrations of a few phosphatidylcholines (PC aa C36:2) were reduced with hepatic lipidosis (Imhasly et al., 2014). Further investigations are necessary to ascertain whether the alterations in some of the glycerolipids are a common or unique feature of metabolic diseases, whether these changes are sustained in the long term, whether resolution of the underlying metabolic disturbances corrects the circulating or urine glycolipid concentrations, and whether these lipid moieties could be utilized as biomarkers for detecting cows susceptible to metabolic diseases.

Hierarchical clustering of the top 25 metabolites showed that the cows fed the anionic salts (negative DCAD diets from both visits 1 and 3) had similar clustering of metabolite classes, which were distinct from those of visit-2 cows fed diets without anionic salts (positive DCAD diet; Figure 2A). For the first component, which contributed to 21% of the total variance in the sPLS-DA scores plot (Figure 2A), the first loading included top 10 variables of which blood base excess, pH, HCO_3_^−^, total CO_2_, and urine concentrations of lysine, carnosine, and glutamine had loadings >0.2 (Figure 2C). The second component contributed to 4% of the total variance in the sPLS-DA scores plot (Figure 2A) and included top 10 variables of which blood lactate, plasma concentrations of lysophosphatidylcholine with acyl residue C14:0, and urine concentrations of C18:2 and arginine had loadings >0.2 (Figure 2C, D). Further studies are necessary to determine whether circulating and urine concentrations of these AA, amines, and lysophophatidylcholines are predictive of cows susceptible to metabolic acidosis, and whether these indices revert to normal after restoration of normal acid-base status.

**Figure 2:**
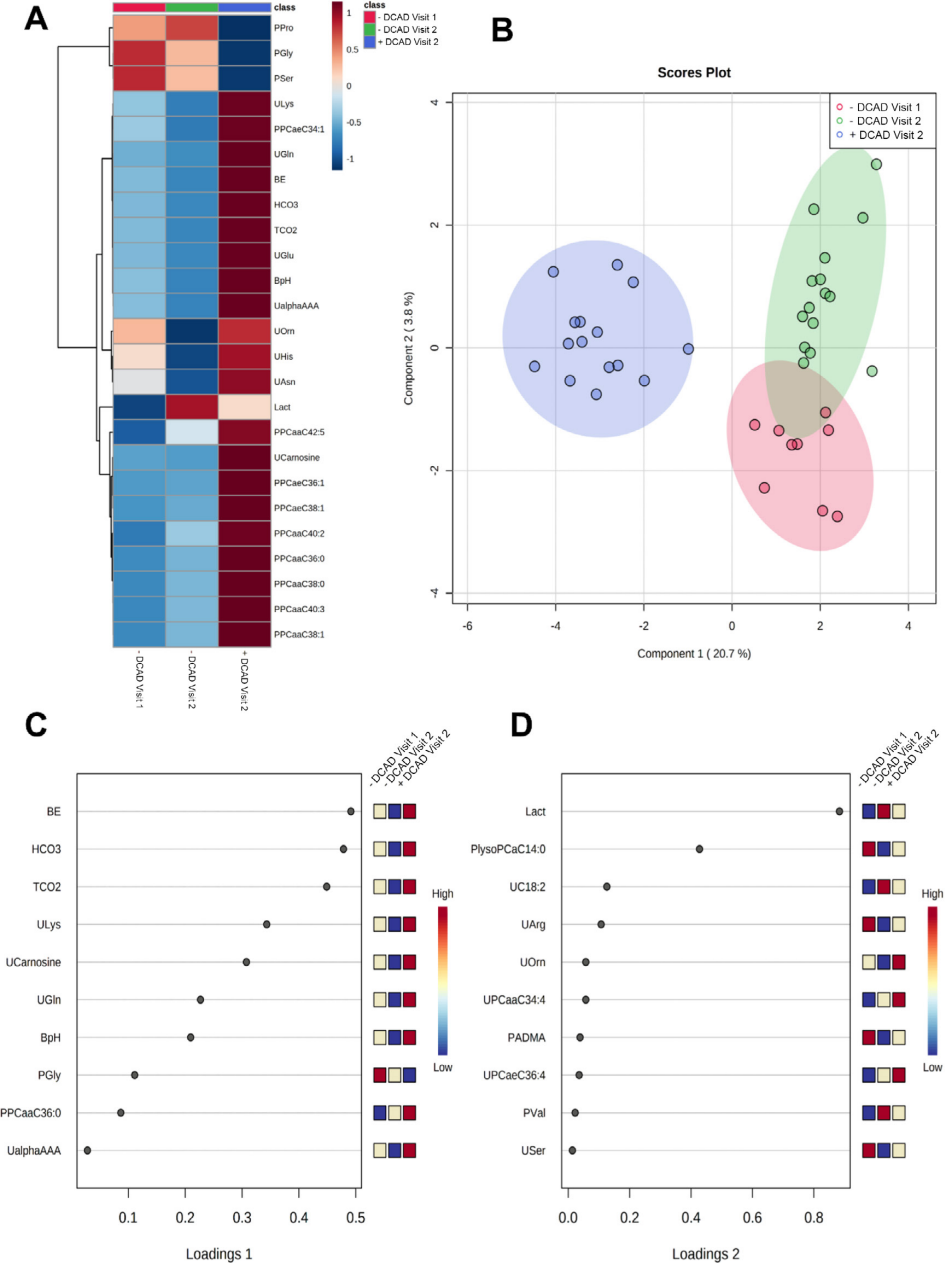
(A) Hierarchical clustering heatmap, (B) scores plot for sparse partial least squares discriminant analyses (sPLS-DA), and associated loadings of the top 10 metabolites from (C) first component and (D) second component, of the physiological and urine and plasma metabolomic data from visit 1, when cows were fed a negative DCAD diet, and in visit 2, when cows were fed an early dry positive DCAD diet, followed by a prepartum negative DCAD diet (visit 3). Metabolites: BE = base excess; HCO3 = bicarbonate, HCO_3_^−^; TCO2 = total CO_2_; ULys = urine lysine; UCarnosine = urine carnosine; UGln = urine glutamine; BpH = blood pH; PGly = plasma glycine; PPCaaC36:0 = plasma phosphatidylcholine with diacyl residue sum C36:0; UalphaAAA = urine α-aminoadipic acid; Lact = lactate; PlysoPCaC14:0 = plasma lysophosphatidylcholine with acyl residue C14:0; UC18:2 = urine C18:2; UArg = urine arginine; UOrn = urine ornithine; UPCaaC34:4 = urine phosphatidylcholine with diacyl residue sum C34:4; PADMA = plasma asymmetric dimethylarginine; UPCaeC36:4 = urine phosphatidylcholine with acyl-alkyl residue sum C36:4; PVal = plasma valine; USer = urine serine.

In the current study, cows consuming the anionic diet had a urine pH <6.0. The normal urine pH for dairy cows, and ruminants in general, is between 8 and 8.5 (Parrah et al., 2013), which is consistent with the urine pH of the early dry cows (not consuming a negative DCAD diet) before entering the prepartum lot. Urine pH values <6.0 may indicate a risk of overacidification and development of a potential uncompensated metabolic acidosis (Goff, 2014; Melendez and Poock, 2017). In fact, blood gas analyses unquestionably demonstrated that cows consuming anionic diets in the current study were experiencing metabolic acidosis. However, the acidosis was severe in most animals because the base excess was negative in all animals consuming anions and below −6 in 14 out of 15 cows (93.3%). The lower the base excess, the more severe the metabolic acidosis (Dillane et al., 2020; van Gastelen et al., 2021). Interestingly, cows consuming the anionic diet had higher concentrations of l-lactate than the same cows eating the regular positive DCAD diet before entering the prepartum group. In a recent study, when dietary DCAD decreased from +110 mEq to −70 mEq/kg of DM and then to −180 mEq/kg of DM, linear decreases in DM intake and blood pH were observed, suggesting that the use of moderately acidogenic diets may benefit dairy cow health and productive performance, but diets that cause an excessive decrease in blood pH or uncompensated metabolic acidosis are more detrimental to the dairy cow (Vieira-Neto et al., 2021a). In our study, it is unclear whether the increase in blood lactate was due to a higher glycolytic flux in peripheral tissues, a potential lower DM intake, or other underlying pathologic mechanism. The reduction in DM intake imposed by anions in negative DCAD diets (Charbonneau et al., 2006) may cause prepartum fat mobilization, with a potential strained use of phosphatidylcholines and impairment of insulin sensitivity, which may reduce tissue uptake of glucose (Bigner et al., 1996). Furthermore, a negative DCAD diet was reported to increase the abundance of proteins involved in lipolysis and decrease the lipogenic enzyme acetyl-CoA carboxylase in adipose tissue (Vieira-Neto et al., 2021b). Finally, one of the unanswered questions in this pilot study was what the metabolomic patterns of urine and plasma would be in cows consuming either a more moderate anionic diet (urine pH >6.0) or a positive DCAD diet, and their metabolic status at calving and during the early postpartum. This comparison would help to reduce the confounding effect imposed by the anionic diet and days in gestation, as in the current study there was no control group with the same days of gestation consuming a positive DCAD diet. However, in a study that compared a prepartum group fed a negative DCAD diet (−130 mEq/kg) and a group consuming a positive DCAD diet (+130 mEq/kg) matched by days of gestation and parity, urine pH was found to be significantly lower (5.7 ± 0.15) in the negative DCAD group than in the positive DCAD group (8.0 ± 0.15; Vieira-Neto et al., 2021b), which was similar to our findings. This suggests that the most important factor affecting the acid-base status of cows was the shift of diets from a positive to a negative DCAD, regardless of differences in days of gestation; whether the stage of pregnancy affected the plasma and urine metabolomics cannot be determined from our study. In summary, we demonstrated that a very low negative DCAD diet leads to uncompensated metabolic acidosis in dairy cows. Importantly, we showed for the first time that such systemic acidosis is associated with distinct alterations in several metabolites in plasma and urine, in particular, glucogenic amino acids, lactic acid, and glycerophospholipids. Further characterization of these metabolomic profiles may lead to the development of novel biomarkers for identifying cows susceptible to metabolic acidosis and other metabolic diseases.
